# Relationship of weight change patterns from young to middle adulthood with incident rheumatoid arthritis and osteoarthritis: a retrospective cohort study

**DOI:** 10.3389/fendo.2023.1308254

**Published:** 2024-01-03

**Authors:** Kai Nan, Ming Zhang, Shouye Hu, Xiaolong Shao, Lin Liu, Yang Zhi, Peng Xu

**Affiliations:** ^1^ Department of Joint Surgery, HongHui Hospital, Xi’an Jiaotong University, Xi’an, Shaanxi, China; ^2^ Department of General Practice, Honghui Hospital, Xi’an Jiaotong University, Xi’an, Shaanxi, China

**Keywords:** weight change, arthritis, rheumatoid arthritis, osteoarthritis, cohort study

## Abstract

**Background:**

The relationship between weight change patterns and arthritis onset, specifically rheumatoid arthritis (RA) and osteoarthritis (OA), is unclear. We examined the association between weight changes from young adulthood to midlife and arthritis onset.

**Methods:**

Using data from NHANES 1999–2018, participants with self-reported arthritis were selected. Age at diagnosis determined arthritis onset. Weight change patterns were based on BMI at age 25 and 10 years before the survey. Patterns were categorized as stable non-obese, non-obese to obese, obese to non-obese, and stable obese. Cox regression models and restricted cubic spline (RCS) analysis were employed, calculating hazard ratios (HRs) and 95% confidence intervals (CIs) considering covariates.

**Results:**

Out of 20,859 participants (male 11,017, 52.82%), 4922 developed arthritis over a mean 8.66-year follow-up. Compared to stable non-obese individuals, the HRs for arthritis were 1.55 (95% CI=1.45 to 1.66, P < 0.0001) for non-obese to obese and 1.74 (95% CI=1.56 to 1.95, P < 0.0001) for stable obese. Those gaining 10-20 kg had a HR of 1.33 (95% CI=1.22 to 1.46, P < 0.0001), and gains >20 kg had a HR of 1.56 (95% CI=1.42 to 1.71, P < 0.0001), compared to stable weight (change within 2.5 kg). Identical results observed for OA and RA. RCS showed a nonlinear relationship between weight change and arthritis (all P < 0.01).

**Conclusions:**

Stable obesity and weight gain during adulthood increase arthritis risk. Maintaining a non-obese weight throughout adult years might reduce arthritis risk in later life.

## 1 Background

Arthritis is a medical condition that involves infections within and around the joints. It is a prevalent chronic systemic inflammatory disease, characterized by symptoms such as redness, swelling, heat, and pain (1). Currently, there are 355 million individuals worldwide who are affected by arthritis (2). It is estimated that by 2040, approximately 25.9% of all American adults will be diagnosed with arthritis (3). This often results in limited movement and a consequent decrease in the quality of life for affected individuals. Its etiology is multifactorial, encompassing genetic, environmental, and lifestyle factors (4). Among these, body weight, particularly as measured by the body mass index (BMI), has emerged as a significant modifiable risk factor for the development of certain types of arthritis, notably osteoarthritis (OA) (5). The Global Burden of Disease study in 2017 highlighted the relevance of high BMI as a primary risk factor for the burden of OA in Brazil (5). This association is not limited to static measures of weight but extends to dynamic changes in weight over time. For instance, an increasing trend of knee radiographs with OA-like features has been observed in rheumatoid arthritis (RA) patients in recent decades, with a higher BMI identified as an independent factor for such manifestations (6).

Of the many types of arthritis, OA and RA are the two most common forms, each affecting millions worldwide and presenting distinct challenges in healthcare (7). While they both share the general term ‘arthritis’, OA and RA have distinct pathophysiological mechanisms, clinical manifestations, and therapeutic approaches (4, 8). OA is a degenerative joint disease primarily affecting the articular cartilage and subchondral bone, often related to aging, mechanical stress, or metabolic disturbances (9). OA is now understood as a disease affecting the entire joint, involving not just the cartilage, but also the subchondral bone, meniscus, synovial membrane, and infrapatellar fat pad (10). It is marked by joint pain, stiffness, and reduced function, predominantly affecting the knees, hips, and hands (11). Conversely, RA is an autoimmune disease leading to chronic inflammation in joints. This systemic disorder is characterized by symmetric polyarthritis, typically affecting the small joints of the hands and feet, and can result in significant joint damage and deformity if not managed properly (4, 12). Additionally, RA is associated with extra-articular manifestations and systemic inflammation, contributing to increased cardiovascular risk and mortality (13). Furthermore, body composition differences between RA and OA patients have been noted, with OA patients typically presenting a higher total fat mass compared to their RA counterparts (14). Besides the substantial physical impact on patients, OA contributes significantly to escalating healthcare costs in the US (15). While there is emerging evidence suggesting that weight loss from young adulthood to midlife can substantially reduce arthritis risk (16), recent studies indicate that the differential effects of weight changes on the onset and progression of OA and RA are complex and warrant further investigation (17).

Thus, understanding these weight change patterns is crucial, as weight trajectories during these life stages can have long-term implications for joint health and overall well-being. Given the profound implications of arthritis and its associated limited activity, addressing this condition remains a pressing concern for both clinical and public health systems. This study aims to shed light on this relationship, offering insights that could inform preventive strategies and interventions.

## 2 Materials and methods

### 2.1 Study population

The National Health and Nutrition Examination Survey (NHANES) is a research project conducted by the National Center for Health Statistics (NCHS) to gather health and nutrition data on the population of the United States. Each year, approximately 5,000 individuals, selected through a nationally representative sample, are included in the survey. To ensure representative results, the organization employs a stratified, multistage, and clustered probability sampling design. Furthermore, all participants provide written informed consent prior to data collection. The study is conducted by qualified medical professionals and staff, who utilize various methods such as questionnaires, physical examinations, and laboratory data available through open online sources (https://www.cdc.gov/nchs/nhanes).

**Figure 1:**
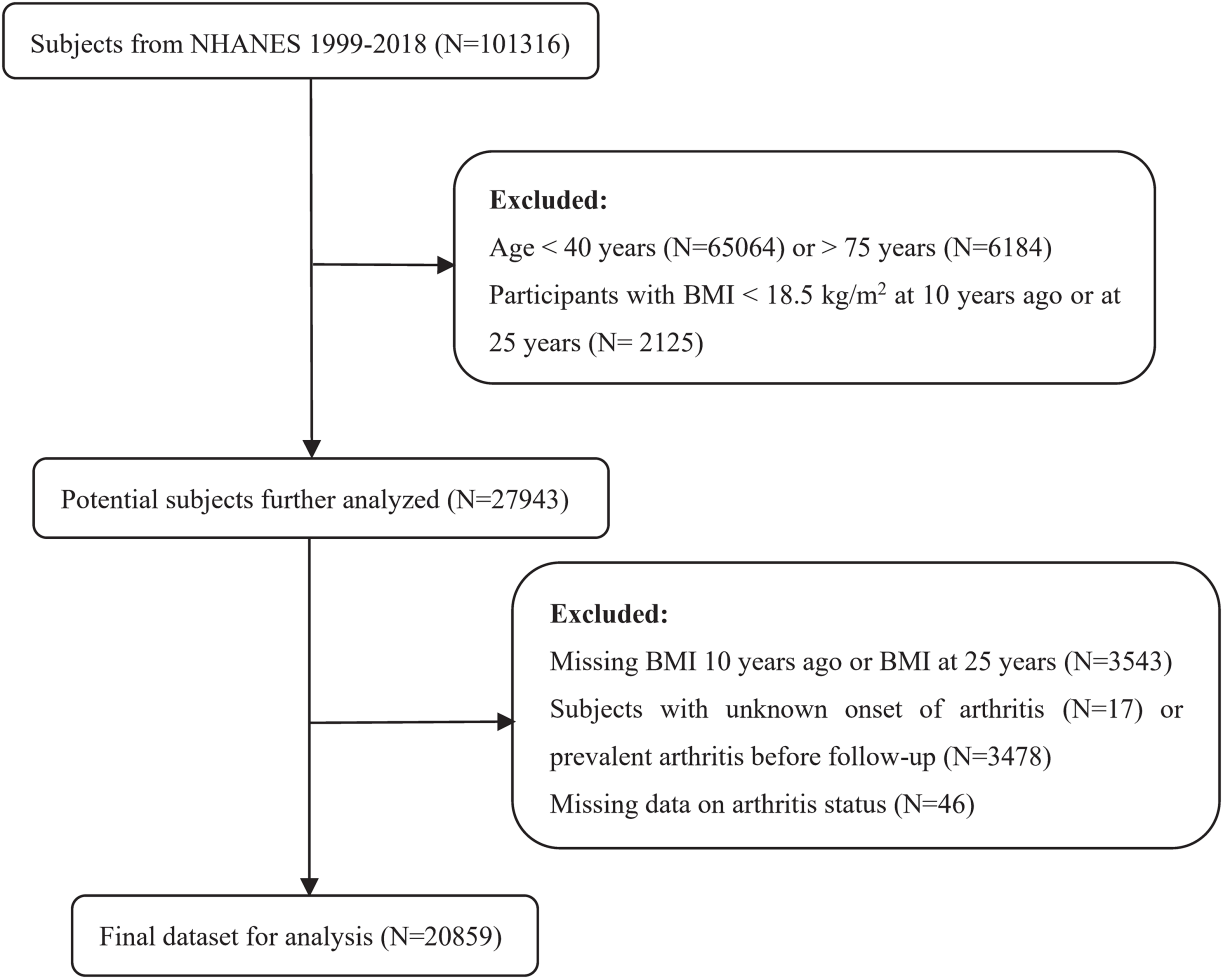
Flow chart of the sample collection in this study.

In this study, data from 10 consecutive NHANES cycles from 1999 to 2018 were used. A total of 101,316 participants were included in the analysis, with exclusions for individuals younger than 40 years or older than 75 years, underweight individuals (BMI 10 years ago or at 25 years < 18.5 kg/m^2^), and those with missing BMI data at 10 years before baseline or at both age 25 years and 10 years before baseline. Moreover, subjects with an unclear onset of arthritis, those who had prevalent arthritis before the beginning of our follow-up study, and individuals with missing information on their arthritis status were excluded. Finally, a total of 20,859 participants were eligible for further analyses. The detailed study flowchart is depicted in **Figure 1**.

### 2.2 Assessment of weight change

The NHANES Anthropometry section utilized standardized examination procedures to measure participants’ standing height and weight at baseline. Participants were asked to recall their weight at age 25 and 10 years prior to the survey, while baseline weight and height were measured during the physical examination. BMI is calculated by taking a person’s weight in kilograms and dividing it by their height in meters squared. Obesity is defined as a BMI of 30.0 kg/m² or higher. BMI was computed based on the measured height during the examination, except for participants aged 50 years and above during the survey. For those aged 50 years or older, their reported height at age 25 was used to determine BMI at that age to account for the possibility of height decline with age, while their measured height during the examination was used to calculate BMI ten years prior to the exam (18). We categorized weight change patterns based on BMI status at the previous two time points, specifically BMI at age 25 and 10 years prior to the survey. These patterns included stable non-obese (BMI < 30 at both times), non-obese to obese (BMI < 30 at age 25 and ≥ 30 ten years before the survey), obesity to non-obesity (BMI ≥ 30 at age 25 and < 30 ten years before the survey), and stable obesity (BMI ≥ 30 at both times). We determined absolute weight change by subtracting weight at age 25 from ten years before the survey. We determined absolute weight change by subtracting weight at age 25 from ten years before the survey. Subsequently, we classified the absolute weight change into five groups: weight loss group (weight loss ≥2.5 kg), stable weight group (weight change within 2.5 kg, reference group), mild weight gain (2.5 kg ≤ weight gain <10.0 kg), moderate weight gain (10 kg ≤ weight gain <20.0 kg), and severe weight gain (weight gain ≥20.0 kg) (19, 20). Covariate data were collected through demographic and questionnaire surveys, which included information on gender, baseline age, ethnicity (Mexican American, non-Hispanic Black, non-Hispanic White, and other race), baseline smoking status (never, former, now), marital status (married, unmarried), history of malignancy (yes or no), baseline education level (less than high school, high school or equivalent, college or above) and baseline family poverty income ratio. Poverty income ratio (0–1.0, 1.1–3.0, > 3.0) was used to reflect family income. Smoking status is divided into three categories: current (individuals who have smoked more than 100 cigarettes in their lifetime and have smoked within the past 2 years at the time of the interview), former (individuals who have smoked more than 100 cigarettes in their lifetime but quit more than 2 years before the interview), and never (individuals who have not smoked 100 cigarettes in their lifetime).

### 2.3 Definitions of OA and RA

Information on arthritis diagnosis is collected from personal interviews where participants self-report their health history. Participants were questioned whether a doctor or other healthcare professional had ever diagnosed them with arthritis. If they responded affirmatively, they were prompted to specify the type of arthritis they had been diagnosed with, such as osteoarthritis (OA), rheumatoid arthritis (RA), psoriatic arthritis, or other forms. We utilized the age participants reported being diagnosed to determine the onset of arthritis. The methodology for this retrospective cohort study, using NHANES data, has been comprehensively described in detail in prior publications (16, 18, 20, 21). The design of the current study is illustrated in **Supplementary Figure S1**.

### 2.4 Statistical analysis

Following the guidelines set by the Centers for Disease Control and Prevention (CDC), we employed complex sample analyses to explore the relationship between weight change patterns and the likelihood of US adults developing arthritis. The NHANES uses design weighting to produce accurate national estimates. Survey-weighted means (95% CI) were used to represent continuous variables between groups, while survey-weighted percentages (95% CI) were used for categorical variables. We used Cox proportional hazards models to determine the hazard ratios (HRs) and their 95% CIs for incident arthritis in relation to weight change patterns from age 25 years to 10 years before survey. For the main analyses, we examined the associations between the four weight change trajectories and developing OA or RA.

The stable non-obesity pattern was used as the reference to which all other weight change patterns were compared. The basic model did not adjust for any variables. In model 1, adjustments were made for baseline age, gender, education status, and race/ethnicity. We further adjusted for marital status, family income-poverty ratio level, smoking status, and history of malignancy in model 2. Using the survival curves corresponding to weight change patterns, we assessed the proportional hazards assumption and found no notable divergence in the proportionality of hazards over time. Subsequent subgroup analyses were then performed based on baseline age (< 50 and≥50 years), gender (male and female), race (Mexican American, non-Hispanic Black, and non-Hispanic White), baseline education level (less than high school, high school or equivalent, college or above), and history of malignancy (yes or no).

We also investigated the associations between absolute weight change groups and arthritis risk. The stable weight group was used as the reference to which all other weight change patterns were compared. Additionally, we examined the dose-response correlation through a restricted cubic spline using four knots at the 5th, 35th, 65th, and 95th. In the restricted cubic spline, the absolute weight changes were also treated as continuous variables. The covariates adjusted within the restricted cubic spline were the same as the covariates adjusted in model 2 of the Cox proportional hazards regression model.

Two sensitivity analyses were performed to test the robustness of the results. These analyses included two key adjustments. First, we excluded participants with a history of malignancy. Recognizing that malignancies can significantly influence an individual’s BMI, either through weight loss (cachexia) or weight gain, depending on the type, location, and stage of the cancer (22, 23), we excluded participants with a history of malignancy. This step aimed to minimize potential confounding effects that these conditions might have on the relationship between BMI changes and arthritis risk. Second, we excluded participants diagnosed with arthritis within the first two years of follow-up to address potential reverse causation bias. This approach helped ensure that the observed associations were not due to pre-existing, undiagnosed arthritis influencing weight changes rather than weight changes influencing the risk of developing arthritis. These sensitivity analyses were critical to validate the stability of our findings and to ensure that the observed associations were not artifacts of specific subgroups or biased by reverse causation. All data processing and analysis were conducted using the R statistical software version 4.1.2 (R Core Team, Vienna, Austria). A p-value of less than 0.05 (two-tailed) was deemed to indicate statistical significance.

## 3 Results

### 3.1 Baseline characteristics

A total of 20,859 participants were included in the study. The prevalence of arthritis was 23.59% (4922/20859) in the overall population. **Table 1** reported weighted characteristics of study participants across weight change patterns from early to middle adulthood. The mean age of the sample was 53.74 years at baseline, and 48.49% were female. The range of age at 10 years before baseline was 30–65 years. The mean BMI was 23.92 kg/m^2^ at age 25, 27.49 kg/m^2^ 10 years before survey, and 29.35 kg/m^2^ at baseline. On average, participants gained 4.93 kg weight from 10 years before survey to baseline. We observed that the distribution of baseline age, gender, race, family poverty income ratio, education level, marital status, BMI at baseline, and BMI 10 years ago were statistically significant (all *P* < 0.05) across weight change patterns. Nevertheless, no significant difference (*P* > 0.05) was observed for smoke status and history of malignancy.

**Table 1:** Characteristics of NHANES 1999–2018 participants based on their weight change trajectories from age 25 to 10 years before survey.

	Stable non-obese	Obese to non-obese	Non-obese to obese	Stable obese	*P*-value
Mean (95% CI) baseline age, years^a^	43.44 (43.17,43.71)	41.01 (39.65,42.38)	46.12 (45.66,46.57)	40.64 (39.95,41.32)	<0.0001
Mean (95% CI) body mass index					
At 10 years before survey	24.90 (24.83,24.98)	26.91 (26.47,27.35)	33.98 (33.77,34.18)	38.70 (38.18,39.22)	<0.0001
At age 25 years	22.57 (22.50,22.63)	33.94 (33.12,34.76)	25.22 (25.09,25.35)	34.44 (34.13,34.75)	<0.0001
Mean (95% CI) absolute weight change, kg	5.90 (5.75,6.06)	-18.69 (-21.53,-15.84)	23.15 (22.53,23.77)	11.23 (9.81,12.64)	<0.0001
Sex					0.0005
Female	49.47 (48.57,50.36)	44.27 (35.53,53.38)	46.58 (44.75,48.42)	43.31 (39.95,46.72)	
Male	50.53 (49.64,51.43)	55.73 (46.62,64.47)	53.42 (51.58,55.25)	56.69 (53.28,60.05)	
Race/ethnicity					<0.0001
Mexican American	5.81 (5.03,6.71)	9.39 (6.31,13.75)	7.07 (5.90,8.45)	7.51 (5.85,9.58)	
Non-Hispanic Black	9.61 (8.63,10.69)	12.19 (8.41,17.36)	11.58 (10.16,13.17)	16.64 (14.04,19.62)	
Non-Hispanic White	73.04 (71.10,74.91)	68.37 (60.75,75.12)	73.53 (70.84,76.05)	68.59 (64.39,72.51)	
Other	11.53 (10.43,12.73)	10.05 (6.38,15.47)	7.82 (6.77,9.03)	7.26 (5.74,9.14)	
Education level^b^					0.0021
Less than high school	14.73 (13.76,15.75)	25.38 (19.03,33.00)	14.66 (13.21,16.24)	14.99 (12.50,17.87)	
High school or equivalent	23.20 (22.17,24.26)	19.93 (13.58,28.28)	25.82 (24.06,27.65)	25.85 (22.27,29.79)	
College or above	62.08 (60.52,63.61)	54.68 (45.26,63.79)	59.52 (57.16,61.84)	59.16 (55.00,63.19)	
PIR^b^					<0.0001
≤1	9.47 (8.72,10.29)	19.63 (14.23,26.45)	9.59 (8.47,10.83)	13.22 (10.89,15.95)	
1.1~3	28.98 (27.59,30.42)	31.83 (23.66,41.30)	33.77 (31.49,36.13)	33.36 (29.79,37.14)	
≥3	61.54 (59.74,63.32)	48.54 (38.73,58.46)	56.64 (53.91,59.34)	53.42 (49.40,57.39)	
History of malignancy^b^					0.8929
No	90.55 (89.89,91.17)	90.04 (83.56,94.15)	90.06 (88.85,91.15)	90.16 (87.75,92.13)	
Yes	9.45 (8.83,10.11)	9.96 (5.85,16.44)	9.94 (8.85,11.15)	9.84 (7.87,12.25)	
Smoker^b^					0.5032
Never	51.10 (49.98,52.22)	47.42 (38.74,56.25)	52.26 (50.12,54.40)	51.75 (48.17,55.30)	
Former	28.88 (27.88,29.90)	33.48 (25.10,43.05)	29.62 (27.73,31.58)	28.28 (24.88,31.94)	
Now	20.02 (19.14,20.92)	19.11 (13.43,26.45)	18.12 (16.76,19.57)	19.97 (17.02,23.30)	
Marital status^b^					<0.0001
Unmarried	27.27 (26.08,28.49)	36.46 (27.99,45.86)	28.84 (27.08,30.67)	34.58 (31.23,38.08)	
Married	72.73 (71.51,73.92)	63.54 (54.14,72.01)	71.16 (69.33,72.92)	65.42 (61.92,68.77)	

For continuous variables: survey-weighted mean (95% CI), P-value was by survey-weighted linear regression (svyglm).

For categorical variables: survey-weighted percentage (95% CI), P-value was by survey-weighted Chi-square test (svytable).

a At start of follow-up.

b At end of follow-up.

### 3.2 Association of weight change patterns and incident OA and RA

**Figure 2:**
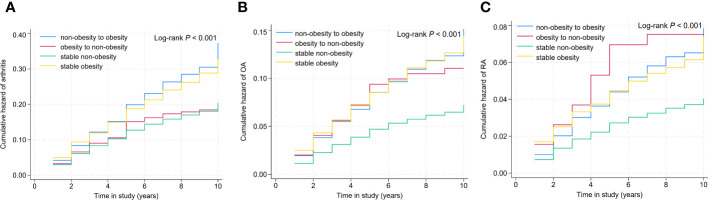
Cumulative incidence curve of arthritis, rheumatoid arthritis, and osteoarthritis for weight change patterns. Arthritis **(A)**, rheumatoid arthritis **(B)**, and osteoarthritis **(C)**.

Among 20,859 participants, 4922 had a diagnosis of arthritis, yielding an overall prevalence rate of 23.59%. **Figure 2** presented cumulative incidence curves by time in study for each weight change group. The cumulative incidence of arthritis, OA, and RA varied significantly among the four weight change patterns, as shown in **Figures 2A–C** (all *P* < 0.01).

Compared with stable non-obesity individuals, non-obese to obese participants had an increased risk of developing arthritis over a 10-year period, with a HR of 1.55 (95% confidence interval [CI]=1.45 to 1.66, *P* < 0.001). Participants who remained obese from young adulthood to midlife had 1.74 times (95% CI: 1.56–1.95) higher risk of developing arthritis during the 10-year follow-up. However, there was no significant difference in arthritis risk (HR 1.02; 95% CI 0.75–1.39) between those who transitioned from an obese BMI to a non-obese BMI during the study period. When considering absolute weight changes, the HRs (95% CI) for arthritis in the moderate weight gain group (10 kg ≤ weight gain < 20.0 kg) were 1.33 (1.22 to 1.46) compared to the stable weight group (weight change within 2.5 kg) from 10 years before the survey to baseline. The HRs (95% CI) for arthritis in the severe weight gain group (weight gain ≥ 20.0 kg) were 1.56 (1.42 to 1.71).

Compared with stable non-obesity individuals, non-obese to obese participants had an increased risk of developing RA over a 10-year period, with a HR of 1.46 (95% CI =1.25 to 1.70, *P* < 0.001). Participants who remained obese from young adulthood to midlife had 1.58 times (95% CI: 1.24–2.00) higher risk of developing RA during the 10-year follow-up. However, the obesity to non-obesity group was not significantly associated with incident RA risk (HR 1.17; 95% CI 0.71–1.93, *P* = 0.5360). When considering absolute weight changes, the HRs (95% CI) for RA in the moderate weight gain group (10 kg ≤ weight gain < 20.0 kg) were 1.32 (1.09 to 1.60) compared to the stable weight group (weight change within 2.5 kg) from 10 years before the survey to baseline. The HRs (95% CI) for RA in the severe weight gain group (weight gain ≥ 20.0 kg) were 1.32 (1.08 to 1.62).

Compared with stable non-obesity individuals, non-obese to obese participants had an increased risk of developing OA over a 10-year period, with a HR of 1.66 (95% CI =1.49 to 1.85, *P* < 0.001). Participants who remained obese from young adulthood to midlife had 1.80 times (95% CI: 1.52–2.13) higher risk of developing OA during the 10-year follow-up. However, the obesity to non-obesity group was not significantly associated with incident OA risk (HR 1.20; 95% CI 0.80–1.79, *P* = 0.3882). When considering absolute weight changes, the HRs (95% CI) for OA in the moderate weight gain group (10 kg ≤ weight gain < 20.0 kg) were 1.32 (1.15 to 1.53) compared to the stable weight group (weight change within 2.5 kg) from 10 years before the survey to baseline. The HRs (95% CI) for OA in the severe weight gain group (weight gain ≥ 20.0 kg) were 1.50 (1.30 to 1.74).

**Figure 3:**
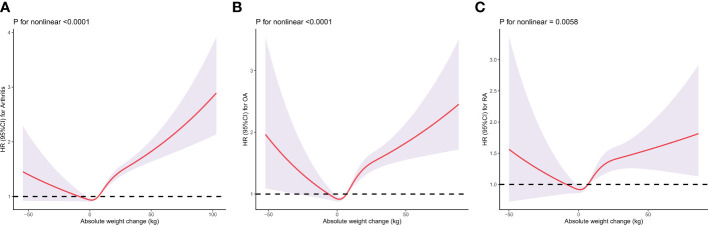
Dose–response association between absolute weight change across adulthood and risk of arthritis **(A)**, rheumatoid arthritis **(B)**, and osteoarthritis **(C)**. Multivariable Cox regression model adjusted for gender, baseline age, race/ethnicity, marital status, education level, family income-poverty ratio level, smoking status, and history of malignancy. Each panel represents a different aspect of arthritis risk: **(A)** for overall arthritis, "**(B)** for OA, and **(C)** for RA. The y-axis indicates the Hazard Ratio (HR) for arthritis, while the x-axis shows the absolute weight change in kilograms. The spline curves demonstrate that the relationship between weight change and arthritis risk is not linear. For example, in **(A)**, there is a notable increase in arthritis risk with both weight gain and weight loss beyond certain thresholds. Similarly, **(B, C)** show distinct patterns for OA and RA, respectively. The ‘P for nonlinear’ values beneath each graph indicates the statistical significance of the nonlinear relationship, with lower values suggesting a stronger departure from linearity.

When evaluating the absolute weight change, there was a U-shaped association between absolute weight change and the risk of incident arthritis, RA and OA (**Figure 3**). After multivariable adjustment, restricted cubic spline showed a U-shaped association between absolute weight change with arthritis (**Figure 3A**), OA (**Figure 3B**), and RA (**Figure 3C**), respectively.

### 3.3 Sensitivity analysis and stratified analyses

**Figure 4:**
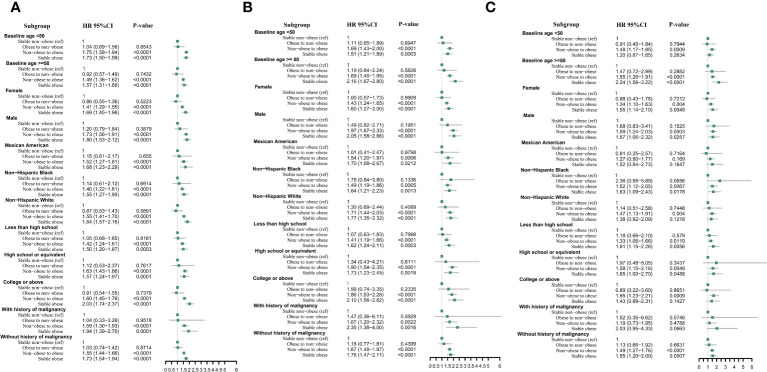
Multivariable cox proportional hazard ratios for arthritis **(A)**, rheumatoid arthritis **(B)**, and osteoarthritis **(C)** with weight change patterns across adulthood in subgroups stratified by patients’ characteristics.

When stratified by baseline age (< 50 and≥50 years), gender (male and female), race (Mexican American, non-Hispanic Black, and non-Hispanic White), baseline education level (less than high school, high school or equivalent, college or above), and history of malignancy (yes or no), the relationships were consistent with our primary findings regarding arthritis, OA, and RA (**Figures 4A–C**).

We conducted additional sensitivity analyses to assess the robustness of our findings. After excluding participants with a history of malignancy, we found that individuals in the non-obese to obese, stable obese, moderate weight gain, and severe weight gain groups had a significantly higher risk of arthritis, OA, and RA even after adjusting for all potential confounders (**Supplementary Table S1**, all *P* < 0.01). Furthermore, when we excluded participants who were diagnosed with arthritis within the first 2 years of follow-up, we still observed a significantly increased risk of arthritis, OA, and RA in the non-obese to obese, stable obese, moderate weight gain, and severe weight gain groups after adjusting for all cofounders (**Supplementary Table S2**, all *P* < 0.01). These results were consistent with the results in **Table 2**.

**Table 2:** Hazard ratio (HR) and 95% confidence intervals (CIs) of arthritis with weight change patterns across adulthood in the NHANES 1999–2018.

	Non-adjusted HR (95%CI), P	Adjust I HR (95%CI), P	Adjust II HR (95%CI), P
Arthritis			
Absolute weight change			
Weight change within 2.5 kg	1 (Reference)	1 (Reference)	1 (Reference)
Weight loss≥2.5 kg	1.13 (0.98, 1.30) 0.1009	1.05 (0.91, 1.21) 0.5306	1.03 (0.89, 1.20) 0.6933
Weight gain≥2.5 kg and < 10 kg	1.11 (1.02, 1.20) 0.0166	1.03 (0.95, 1.12) 0.4609	1.02 (0.94, 1.12) 0.6268
Weight gain≥10 kg and < 20 kg	1.60 (1.47, 1.74) <0.0001	1.34 (1.24, 1.46) <0.0001	1.33 (1.22, 1.46) <0.0001
Weight gain≥20 kg	2.04 (1.87, 2.23) <0.0001	1.59 (1.45, 1.74) <0.0001	1.56 (1.42, 1.71) <0.0001
Weight change patterns			
Stable non-obese	1 (Reference)	1 (Reference)	1 (Reference)
Obese to non-obese	0.99 (0.73, 1.33) 0.9347	1.06 (0.79, 1.43) 0.6842	1.02 (0.75, 1.39) 0.8872
Non-obese to obese	1.77 (1.67, 1.89) <0.0001	1.58 (1.48, 1.68) <0.0001	1.55 (1.45, 1.66) <0.0001
Stable obese	1.57 (1.41, 1.74) <0.0001	1.76 (1.59, 1.96) <0.0001	1.74 (1.56, 1.95) <0.0001
Osteoarthritis			
Absolute weight change			
Weight change within 2.5 kg	1 (Reference)	1 (Reference)	1 (Reference)
Weight loss≥2.5 kg	1.17 (0.94, 1.47) 0.1654	1.07 (0.85, 1.34) 0.5539	0.96 (0.77, 1.21) 0.7501
Weight gain≥2.5 kg and < 10 kg	0.86 (0.75, 0.99) 0.0341	0.83 (0.72, 0.96) 0.0101	0.91 (0.79, 1.04) 0.1679
Weight gain≥10 kg and < 20 kg	1.39 (1.21, 1.60) <0.0001	1.21 (1.05, 1.39) 0.0080	1.32 (1.15, 1.53) <0.0001
Weight gain≥20 kg	1.96 (1.70, 2.26) <0.0001	1.54 (1.33, 1.78) <0.0001	1.50 (1.30, 1.74) <0.0001
Weight change patterns			
Stable non-obese	1 (Reference)	1 (Reference)	1 (Reference)
Obese to non-obese	1.60 (1.07, 2.40) 0.0232	1.47 (0.98, 2.21) 0.0616	1.20 (0.80, 1.79) 0.3882
Non-obese to obese	2.07 (1.86, 2.31) <0.0001	1.81 (1.63, 2.02) <0.0001	1.66 (1.49, 1.85) <0.0001
Stable obese	1.94 (1.64, 2.30) <0.0001	2.03 (1.72, 2.41) <0.0001	1.80 (1.52, 2.13) <0.0001
Rheumatoid arthritis			
Absolute weight change			
Weight change within 2.5 kg	1 (Reference)	1 (Reference)	1 (Reference)
Weight loss≥2.5 kg	1.08 (0.79, 1.47) 0.6314	0.98 (0.72, 1.34) 0.9141	0.87 (0.63, 1.18) 0.3702
Weight gain≥2.5 kg and < 10 kg	0.83 (0.69, 1.00) 0.0469	0.81 (0.67, 0.98) 0.0306	0.90 (0.75, 1.09) 0.2944
Weight gain≥10 kg and < 20 kg	1.27 (1.05, 1.54) 0.0118	1.13 (0.94, 1.37) 0.1994	1.32 (1.09, 1.60) 0.0048
Weight gain≥20 kg	1.72 (1.41, 2.10) <0.0001	1.37 (1.12, 1.68) 0.0024	1.32 (1.08, 1.62) 0.0074
Weight change patterns			
Stable non-obese	1 (Reference)	1 (Reference)	1 (Reference)
Obese to non-obese	1.95 (1.19, 3.21) 0.0083	1.69 (1.03, 2.78) 0.0396	1.17 (0.71, 1.93) 0.5360
Non-obese to obese	1.96 (1.68, 2.27) <0.0001	1.71 (1.47, 1.99) <0.0001	1.46 (1.25, 1.70) <0.0001
Stable obese	1.83 (1.45, 2.32) <0.0001	1.84 (1.45, 2.34) <0.0001	1.58 (1.24, 2.00) 0.0002

Non-adjusted model adjust for: None.

Adjust I model adjust for: sex, race, education level, baseline age.

Adjust II model adjust for: sex, race, education level, baseline age, marital status, smoke status, family poverty income ratio, and history of malignancy.

## 4 Discussion

Based on the nationally representative US adults’ cohort, we revealed the association between long-term weight change patterns across adulthood and arthritis, OA, and RA. Our study, which utilizes the NHANES database, provides insight into the intricate pathogenesis of arthritis by exploring the relationship between weight fluctuations during various life stages and the subsequent risk of joint diseases. Our findings highlight a significant correlation between changes in weight from early adulthood to middle age and the likelihood of developing both OA and RA.

This interplay between weight dynamics and arthritis risk can be understood better through several underlying mechanisms. Excess weight places excessive stress on weight-bearing joints, making individuals more susceptible to OA (24). This increased mechanical stress, combined with changes in gait and biomechanics, can accelerate joint degeneration (25). In addition to these mechanical factors, systemic inflammation plays a crucial role in the development of both RA and OA. Adipose tissue actively produces inflammatory mediators such as TNF-alpha and IL-6 (26). Additionally, the role of the infrapatellar fat pad (IPFP) in OA deserves attention. Recent studies have identified the IPFP as an active mediator in OA pathogenesis, with its inflammation and hypertrophy linked to increased OA severity (27). This highlights the complex interplay between adipose tissue within the joint and OA development. Leptin, an adipokine, has implications beyond regulating appetite, contributing to cartilage degradation and inflammation (28). Furthermore, changes in diet and weight can affect the gut microbiome, which may impact systemic inflammation and conditions like RA (29). Disparities in gut microbial equilibrium can potentiate systemic inflammation, affecting distal organs, including the joints (30).

Perspectives on BMI and arthritis vary. Some cohort studies deduced childhood overweight metrics to associate with adult knee pain, specifically among men, irrespective of adult weight (31). On the other hand, some studies have shown that there is no clear association between adolescent BMI and the risk of knee osteoarthritis when adult BMI is taken into account (32). Our analysis also supports these findings by demonstrating that individuals who undergo weight loss over the course of their lifetime have similar risks of developing arthritis as those who maintain a consistently lower weight. Our findings concur with previous epidemiological studies that have linked obesity with the onset of arthritis (33). Obesity has long been recognized as a major risk factor for OA, particularly of the knee (34). The mechanical strain exerted by excess weight on the weight-bearing joints is thought to contribute to cartilage breakdown, the hallmark of OA (35). Advancements in biomechanics highlight the crucial role of mechanical strain in OA pathogenesis, particularly in cartilage breakdown. Chondrocytes, central to articular cartilage, respond significantly to mechanical loading within the extracellular matrix. This response influences cartilage integrity and OA progression. Studies emphasize that understanding chondrocyte biomechanics is key to comprehending OA development, linking mechanical properties and cellular responses to OA’s onset and severity (36, 37). However, the relationship between obesity and RA has been less consistently established. A meta-analysis found a modestly increased risk of RA in obese individuals, suggesting that adipose tissue might play a role in RA’s pathogenesis by producing pro-inflammatory cytokines (38). Our study provides further insight into this topic by examining the risk of arthritis based on patterns of weight change. We found that individuals who transitioned from a non-obese state to an obese state had a higher hazard ratio for developing arthritis than those who maintained a stable non-obese weight. This progression towards obesity over time may expose individuals to prolonged periods of systemic inflammation, thereby increasing their risk of developing both RA and OA (39). It’s interesting that participants in the “obese to non-obese” category were not significantly associated with an increased risk in our study. This underscores the potential reversibility of some obesity-related risks with weight reduction. To clarify, this observation indicates that individuals who were initially obese but later transitioned to a non-obese status did not demonstrate the same elevated risk for arthritis as those who remained obese or those who transitioned from non-obese to obese. This finding suggests a notable aspect of our study: the potential for mitigating obesity-related arthritis risks through weight loss. It implies that individuals who successfully reduce their weight from an obese to a non-obese BMI might lower or even negate the heightened arthritis risk typically associated with obesity. This could be due to improvements in systemic inflammation, mechanical stress on joints, or other factors related to weight change. Our analysis thus highlights the importance of weight management and its possible protective effects against arthritis risk. Although weight loss interventions have been shown to reduce the symptoms and progression of established OA (40), our findings suggest that weight loss could also have preventive implications for those at risk. Furthermore, the observed nonlinear relationship between absolute weight change and arthritis risk is of interest. This could indicate that small amounts of weight gain might not significantly influence arthritis risk, but more pronounced weight changes have substantial impacts.

The strengths of our study include the utilization of a nationally representative database, which enhances the generalizability of our findings. Furthermore, by categorizing participants based on weight change patterns, we could investigate the risk associated with dynamic weight changes, which is more representative of real-life scenarios than static BMI measurements at a single point in time. Our findings indicate significant associations between weight change patterns and arthritis risk. However, it is essential to acknowledge that these correlations do not inherently imply causation. The observational nature of our study limits our ability to establish direct causal relationships. We recognize potential confounding factors, such as lifestyle behaviors, genetic predispositions, and other comorbidities, which were not fully controlled in our analysis. Additionally, the reliance on self-reported data in NHANES might introduce recall bias, particularly regarding arthritis diagnosis and historical weight information. These considerations highlight the need for cautious interpretation of our results and underscore the importance of further research to elucidate the complex interplay between weight dynamics and arthritis risk.

## 5 Conclusions

Our study highlights the importance of weight management throughout adulthood as a potentially effective strategy for preventing the onset of arthritis, including its major subtypes, osteoarthritis and rheumatoid arthritis. As obesity and arthritis continue to increase in prevalence globally, our findings highlight the need for public health initiatives that prioritize weight stability and reduction from early adulthood onwards. Future prospective studies are needed to further investigate the mechanisms underlying the relationship between weight change patterns and arthritis risk, and to explore potential interventions that could mitigate this risk.

## Data availability statement

Publicly available datasets were analyzed in this study. This data can be found here: Publicly available dataset was analyzed in this study. The National Health and Nutrition Examination Survey dataset are publicly available at https://www.cdc.gov/nchs/nhanes/index.htm.

## Author contributions

KN: Data curation, Formal Analysis, Validation, Writing – original draft, Writing – review & editing. MZ: Conceptualization, Data curation, Formal Analysis, Software, Validation, Writing – original draft, Writing – review & editing. SH: Data curation, Investigation, Methodology, Resources, Software, Visualization, Writing – original draft. XS: Conceptualization, Formal analysis, Methodology, Resources, Visualization, Writing – original draft, Writing – review & editing. LL: Data curation, Methodology, Project administration, Resources, Supervision, Visualization, Writing – review & editing. YZ: Data curation, Funding acquisition, Resources, Software, Supervision, Visualization, Writing – original draft. PX: Data curation, Funding acquisition, Investigation, Project administration, Supervision, Validation, Visualization, Writing – review & editing.

## Supplementary material

Supplementary Figure 1 Survival analysis study design: weight change and arthritis onset.
