# Supplementary material for: Relationship of weight change patterns from young to middle adulthood with incident rheumatoid arthritis and osteoarthritis: a retrospective cohort study
Source: Front Endocrinol (Lausanne). 2024 Jan 3;14:1308254. doi: 10.3389/fendo.2023.1308254 (PMC10791826; doi:10.3389/fendo.2023.1308254)
Supplement: Supplementary Figure 1 — Survival analysis study design: weight change and arthritis onset. [file DataSheet_1.pdf]

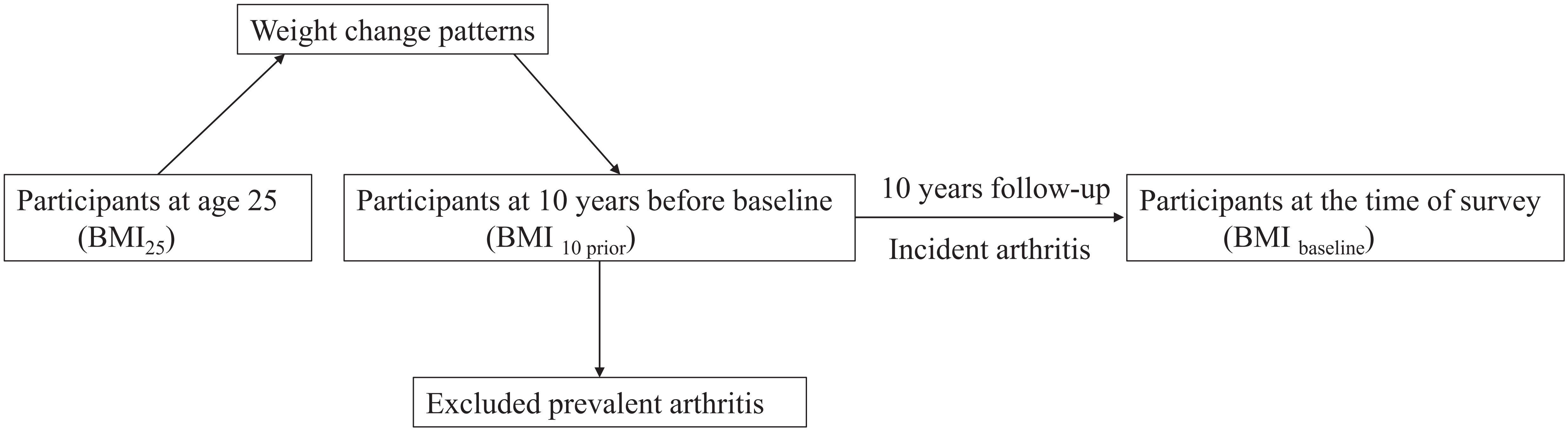
Weight change patterns
Participants at age 25
(BMI25)
Participants at 10 years before baseline
(BMI 10 prior)
10 years follow-up
Incident arthritis
Participants at the time of survey
(BMI baseline)
Excluded prevalent arthritis
