# Supplementary material for: Relationship of weight change patterns from young to middle adulthood with incident rheumatoid arthritis and osteoarthritis: a retrospective cohort study
Source: Front Endocrinol (Lausanne). 2024 Jan 3;14:1308254. doi: 10.3389/fendo.2023.1308254 (PMC10791826; doi:10.3389/fendo.2023.1308254)
Supplement: Supplementary file 2 [file Table_1.docx]

**Table S1** Sensitivity analyses after excluded participants with history of malignancy

|  | Non-adjusted HR (95%CI), P | Adjust I HR (95%CI), P | Adjust II HR (95%CI), P |
| --- | --- | --- | --- |
| Arthritis |  |  |  |
| Absolute weight change |  |  |  |
| Weight change within 2.5 kg | 1 (Reference) | 1 (Reference) | 1 (Reference) |
| Weight loss≥2.5 kg | 1.13 (0.97, 1.32) 0.1069 | 1.05 (0.90, 1.22) 0.5381 | 1.03 (0.88, 1.21) 0.6869 |
| Weight gain≥2.5 kg and < 10 kg | 1.11 (1.02, 1.22) 0.0170 | 1.03 (0.94, 1.13) 0.4939 | 1.02 (0.93, 1.11) 0.7420 |
| Weight gain≥10 kg and < 20 kg | 1.57 (1.44, 1.72) <0.0001 | 1.32 (1.20, 1.44) <0.0001 | 1.30 (1.18, 1.43) <0.0001 |
| Weight gain≥20 kg | 2.02 (1.84, 2.22) <0.0001 | 1.57 (1.43, 1.73) <0.0001 | 1.54 (1.39, 1.70) <0.0001 |
| Weight change patterns |  |  |  |
| Stable non-obese | 1 (Reference) | 1 (Reference) | 1 (Reference) |
| Obese to non-obese | 1.02 (0.75, 1.38) 0.9207 | 1.08 (0.79, 1.47) 0.6150 | 1.03 (0.74, 1.42) 0.8714 |
| Non-obese to obese | 1.76 (1.65, 1.88) <0.0001 | 1.58 (1.47, 1.69) <0.0001 | 1.55 (1.44, 1.66) <0.0001 |
| Stable obese | 1.56 (1.39, 1.74) <0.0001 | 1.75 (1.56, 1.95) <0.0001 | 1.73 (1.54, 1.94) <0.0001 |
| Osteoarthritis |  |  |  |
| Absolute weight change |  |  |  |
| Weight change within 2.5 kg | 1 (Reference) | 1 (Reference) | 1 (Reference) |
| Weight loss≥2.5 kg | 1.13 (0.89, 1.44) 0.3098 | 1.03 (0.81, 1.31) 0.8182 | 0.91 (0.71, 1.16) 0.4403 |
| Weight gain≥2.5 kg and < 10 kg | 0.84 (0.73, 0.97) 0.0214 | 0.81 (0.70, 0.94) 0.0059 | 0.87 (0.75, 1.02) 0.0778 |
| Weight gain≥10 kg and < 20 kg | 1.33 (1.14, 1.54) 0.0002 | 1.15 (0.99, 1.34) 0.0672 | 1.27 (1.09, 1.48) 0.0017 |
| Weight gain≥20 kg | 1.90 (1.63, 2.21) <0.0001 | 1.51 (1.29, 1.76) <0.0001 | 1.47 (1.26, 1.71) <0.0001 |
| Weight change patterns |  |  |  |
| Stable non-obese | 1 (Reference) | 1 (Reference) | 1 (Reference) |
| Obese to non-obese | 1.63 (1.07, 2.48) 0.0243 | 1.47 (0.96, 2.25) 0.0737 | 1.18 (0.77, 1.81) 0.4399 |
| Non-obese to obese | 2.05 (1.83, 2.30) <0.0001 | 1.81 (1.61, 2.03) <0.0001 | 1.67 (1.48, 1.87) <0.0001 |
| Stable obese | 1.95 (1.63, 2.32) <0.0001 | 2.03 (1.70, 2.43) <0.0001 | 1.76 (1.47, 2.11) <0.0001 |
| Rheumatoid arthritis |  |  |  |
| Absolute weight change |  |  |  |
| Weight change within 2.5 kg | 1 (Reference) | 1 (Reference) | 1 (Reference) |
| Weight loss≥2.5 kg | 1.08 (0.78, 1.51) 0.6333 | 0.98 (0.70, 1.37) 0.9135 | 0.83 (0.59, 1.16) 0.2672 |
| Weight gain≥2.5 kg and < 10 kg | 0.85 (0.69, 1.03) 0.0995 | 0.83 (0.68, 1.02) 0.0743 | 0.91 (0.75, 1.12) 0.3709 |
| Weight gain≥10 kg and < 20 kg | 1.26 (1.03, 1.54) 0.0262 | 1.12 (0.91, 1.38) 0.2758 | 1.32 (1.07, 1.62) 0.0094 |
| Weight gain≥20 kg | 1.72 (1.39, 2.13) <0.0001 | 1.39 (1.12, 1.73) 0.0028 | 1.34 (1.08, 1.67) 0.0076 |
| Weight change patterns |  |  |  |
| Stable non-obese | 1 (Reference) | 1 (Reference) | 1 (Reference) |
| Obese to non-obese | 1.91 (1.12, 3.25) 0.0170 | 1.62 (0.95, 2.76) 0.0748 | 1.13 (0.66, 1.92) 0.6631 |
| Non-obese to obese | 1.93 (1.65, 2.27) <0.0001 | 1.73 (1.47, 2.03) <0.0001 | 1.49 (1.27, 1.76) <0.0001 |
| Stable obese | 1.84 (1.43, 2.36) <0.0001 | 1.85 (1.44, 2.38) <0.0001 | 1.55 (1.20, 2.00) 0.0007 |

Non-adjusted model adjust for: None

Adjust I model adjust for: sex, race, education level, baseline age

Adjust II model adjust for: sex, race, education level, baseline age, marital status, smoke status, family poverty income ratio, and history of malignancy
