# Supplementary material for: Relationship of weight change patterns from young to middle adulthood with incident rheumatoid arthritis and osteoarthritis: a retrospective cohort study
Source: Front Endocrinol (Lausanne). 2024 Jan 3;14:1308254. doi: 10.3389/fendo.2023.1308254 (PMC10791826; doi:10.3389/fendo.2023.1308254)
Supplement: Supplementary file 3 [file Table_2.docx]

**Table S2** Sensitivity analyses after excluded participants who diagnosed with arthritis within 2 years of follow-up

|  | Non-adjusted HR (95%CI), P | Adjust I HR (95%CI), P | Adjust II HR (95%CI), P |
| --- | --- | --- | --- |
| Arthritis |  |  |  |
| Absolute weight change |  |  |  |
| Weight change within 2.5 kg | 1 (Reference) | 1 (Reference) | 1 (Reference) |
| Weight loss≥2.5 kg | 1.21 (1.01, 1.45) 0.0379 | 1.11 (0.93, 1.34) 0.2411 | 1.08 (0.89, 1.30) 0.4329 |
| Weight gain≥2.5 kg and < 10 kg | 1.11 (1.00, 1.24) 0.0490 | 1.03 (0.92, 1.14) 0.6300 | 1.00 (0.90, 1.12) 0.9411 |
| Weight gain≥10 kg and < 20 kg | 1.73 (1.55, 1.92) <0.0001 | 1.42 (1.27, 1.58) <0.0001 | 1.40 (1.25, 1.57) <0.0001 |
| Weight gain≥20 kg | 2.31 (2.07, 2.57) <0.0001 | 1.73 (1.55, 1.94) <0.0001 | 1.69 (1.51, 1.90) <0.0001 |
| Weight change patterns |  |  |  |
| Stable non-obese | 1 (Reference) | 1 (Reference) | 1 (Reference) |
| Obese to non-obese | 0.92 (0.62, 1.36) 0.6778 | 1.01 (0.68, 1.50) 0.9649 | 1.00 (0.67, 1.50) 0.9982 |
| Non-obese to obese | 2.00 (1.85, 2.16) <0.0001 | 1.75 (1.62, 1.90) <0.0001 | 1.72 (1.58, 1.87) <0.0001 |
| Stable obese | 1.62 (1.42, 1.85) <0.0001 | 1.88 (1.64, 2.15) <0.0001 | 1.86 (1.62, 2.14) <0.0001 |
| Osteoarthritis |  |  |  |
| Absolute weight change |  |  |  |
| Weight change within 2.5 kg | 1 (Reference) | 1 (Reference) | 1 (Reference) |
| Weight loss≥2.5 kg | 1.16 (0.87, 1.54) 0.3063 | 1.05 (0.79, 1.40) 0.7184 | 0.94 (0.71, 1.26) 0.6938 |
| Weight gain≥2.5 kg and < 10 kg | 0.80 (0.67, 0.96) 0.0162 | 0.77 (0.65, 0.92) 0.0046 | 0.83 (0.69, 0.99) 0.0408 |
| Weight gain≥10 kg and < 20 kg | 1.44 (1.21, 1.71) <0.0001 | 1.24 (1.04, 1.48) 0.0157 | 1.38 (1.16, 1.65) 0.0003 |
| Weight gain≥20 kg | 1.95 (1.63, 2.34) <0.0001 | 1.52 (1.26, 1.82) <0.0001 | 1.48 (1.23, 1.78) <0.0001 |
| Weight change patterns |  |  |  |
| Stable non-obese | 1 (Reference) | 1 (Reference) | 1 (Reference) |
| Obese to non-obese | 1.43 (0.82, 2.47) 0.2033 | 1.34 (0.77, 2.31) 0.3028 | 1.10 (0.63, 1.91) 0.7383 |
| Non-obese to obese | 2.27 (1.99, 2.59) <0.0001 | 1.99 (1.74, 2.28) <0.0001 | 1.83 (1.60, 2.09) <0.0001 |
| Stable obese | 2.04 (1.65, 2.52) <0.0001 | 2.20 (1.78, 2.73) <0.0001 | 1.96 (1.58, 2.43) <0.0001 |
| Rheumatoid arthritis |  |  |  |
| Absolute weight change |  |  |  |
| Weight change within 2.5 kg | 1 (Reference) | 1 (Reference) | 1 (Reference) |
| Weight loss≥2.5 kg | 1.23 (0.84, 1.80) 0.2986 | 1.11 (0.75, 1.62) 0.6039 | 0.99 (0.67, 1.45) 0.9429 |
| Weight gain≥2.5 kg and < 10 kg | 0.84 (0.66, 1.07) 0.1607 | 0.81 (0.64, 1.04) 0.0966 | 0.89 (0.70, 1.14) 0.3525 |
| Weight gain≥10 kg and < 20 kg | 1.34 (1.05, 1.71) 0.0171 | 1.17 (0.91, 1.49) 0.2139 | 1.41 (1.10, 1.81) 0.0061 |
| Weight gain≥20 kg | 1.83 (1.42, 2.36) <0.0001 | 1.43 (1.11, 1.85) 0.0063 | 1.41 (1.08, 1.82) 0.0101 |
| Weight change patterns |  |  |  |
| Stable non-obese | 1 (Reference) | 1 (Reference) | 1 (Reference) |
| Obese to non-obese | 1.85 (0.96, 3.59) 0.0678 | 1.63 (0.84, 3.16) 0.1496 | 1.15 (0.59, 2.25) 0.6732 |
| Non-obese to obese | 2.17 (1.80, 2.62) <0.0001 | 1.91 (1.58, 2.30) <0.0001 | 1.63 (1.35, 1.97) <0.0001 |
| Stable obese | 1.83 (1.35, 2.48) 0.0001 | 1.91 (1.40, 2.60) <0.0001 | 1.67 (1.22, 2.28) 0.0013 |

Non-adjusted model adjust for: None

Adjust I model adjust for: sex, race, education level, baseline age

Adjust II model adjust for: sex, race, education level, baseline age, marital status, smoke status, family poverty income ratio, and history of malignancy
